# Exploring the perceptions of patients with chronic respiratory diseases and their insights into pulmonary rehabilitation in Bangladesh

**DOI:** 10.7189/jogh.14.04036

**Published:** 2024-02-02

**Authors:** GM Monsur Habib, Nazim Uzzaman, Roberto Rabinovich, Sumaiya Akhter, Mohsin Ali, Mustarin Sultana, Hilary Pinnock, RESPIRE Collaboration

**Affiliations:** 1 Community Respiratory Centre, Khulna, Bangladesh Primary Care Respiratory Society (BPCRS), Bangladesh; 2 ELEGI/Colt laboratory, Centre for Inflammation Research, Queen's Medical Research Institute (QMRI), University of Edinburgh, Edinburgh, UK; 3 NIHR Global Health Research Unit on Respiratory Health (RESPIRE), Usher Institute, University of Edinburgh, Edinburgh, UK

## Abstract

**Background:**

Chronic respiratory diseases (CRDs) require holistic management which considers patients’ preferences, appropriate pharmacotherapy, pulmonary rehabilitation, and integrated care. We aimed to understand the perceptions of people with CRDs about their condition and pulmonary rehabilitation in Bangladesh.

**Methods:**

We conducted semi-structured interviews with a maximum variation sample of people with CRDs who had participated in a feasibility study of pulmonary rehabilitation in 2021/2022. A multidisciplinary team transcribed the interviews verbatim and analysed them in Bengali using a grounded theory approach.

**Results:**

We interviewed 15 participants with chronic obstructive pulmonary disease, asthma, or post-tuberculosis. The analysis revealed three themes. The first encompassed understanding CRDs: Patients characterised their condition by the symptoms (e.g. ‘Hapani’ meaning ‘breathlessness’) rather than describing a disease entity. Some believed occupation, previous infection, or family history to be a cause. The second theme included perceptions of pulmonary rehabilitation: Exercise was counterintuitive, as it exacerbated the breathlessness symptom that defined their disease. Views varied, though many acknowledged the benefits after a few sessions. Even with home-based programmes, participants described practical barriers to finding time for the sessions and adopted strategies to overcome the challenges. The third theme focused on implementation: Participants highlighted the need for raising awareness of CRDs and the potential of pulmonary rehabilitation in the community, adapting to the local context, and establishing an accessible resourced service.

**Conclusions:**

Understanding how patients and their communities perceive their condition and the barriers (both conceptual and logistical) to acceptance is the first step to embedding this highly effective intervention into routine health care services in Bangladesh with potential benefits for the increasing number of people living with CRDs in low- and middle-income countries.

The prevalence of chronic respiratory diseases (CRDs) in Bangladesh is increasing [1,2]. Their severity, types, and spectrum in low- and middle-income countries (LMICs) differ from those in developed countries [3]. While there are no high-quality estimates of CRDs prevalence in Bangladesh, some studies suggest that there are approximately 10 million people with asthma, six million with chronic obstructive pulmonary disease (COPD), and a substantial minority with pulmonary impairment after tuberculosis (PIAT), bronchiectasis, interstitial lung disease (ILD), and other, often undiagnosed lung diseases causing significant breathlessness [4,5].

Globally recognised management approaches for CRDs encompass disease-specific pharmacotherapy for both acute and stable conditions, empowering patients through education and supporting self-management, pulmonary rehabilitation, and integrated health care [6]. However, the health care system of Bangladesh, similar to that of other LMICs, prioritises symptom management and the treatment of acute exacerbations for CRDs [7], while effective interventions to improve quality of life and prevent acute exacerbations such as pulmonary rehabilitation [8-12] remain underutilised and difficult to access. Pulmonary rehabilitation centres are scarce, as are skilled professionals able to deliver pulmonary rehabilitation services [13,14].

Understanding patients' perceptions, beliefs, and behaviours regarding their disease is essential for effective disease management, as they influence treatment adherence, self-management practices, treatment decision-making, and psychological well-being [15,16]. Patient-centred care that reflects preferences improves health outcomes and patient satisfaction [17]. In our previous work, stakeholders recommended evaluating the local context and fostering community understanding as crucial steps in implementing effective management of CRDs [13].

There has yet been no qualitative study on CRDs beliefs and management including PR in the context of Bangladesh. We sought to address this gap by understanding the beliefs held by patients with CRDs about their condition; exploring their perceptions of pulmonary rehabilitation as an integral part of their management strategy; and gaining insights into how patients consider that pulmonary rehabilitation could be implemented in Bangladesh.

## METHODS

We conducted this study between 2021 and 2022, with ethical approval from the University of Edinburgh Research Ethics Committee (Reference: 2019-045-ER) and the BRAC James P Grant School of Public Health, BRAC University in Bangladesh (Reference: 2019-045-ER). Sponsorship approval was from the Academic and Clinical Central Office for Research and Development (Reference: AC 200004). We followed the Standards for Reporting Qualitative Research (SRQR) checklist [18] in reporting this study (Table S1 in the **Online Supplementary Document**).

### Study setting

The study took place at a community-based pulmonary rehabilitation centre in Khulna, one of three operated by the Bangladesh Primary Care Respiratory Society. This centre is equipped with facilities for patient assessment, exercise training, educational resources for patients, and other essential tools, all in line with a low-resource model. Operating six days a week, the centre provides consultations to approximately 30–40 patients with CRDs each day.

### Identification and recruitment of the participants

A pulmonary rehabilitation programme was held at the Khulna Centre as part of a mixed-method feasibility study for adults aged ≥18 with CRDs; initially designed as a centre-based programme, it was suspended due to the onset of the coronavirus disease 2019 (COVID-19) pandemic. At that time, the first group of 10 participants completed 70% of the pulmonary rehabilitation sessions. When we were allowed to recommence the feasibility study, we switched to a home-based programme and recruited a further 51 participants.

For this qualitative study, we recruited a purposive sample of participants who had completed the home-based programme or who had participated in the pre-pandemic centre-based programme, representing a broad range of sex, age, distance between home and the Centre, disease and duration of CRDs, and severity. We recruited patients until data saturation was reached (i.e. no additional pertinent information was emerging related to our key objectives). Before seeking consent, we provided the participants with an information sheet outlining all relevant details and answered any of their related questions.

### Development of interview guides

Building on our previous systematic review of pulmonary rehabilitation in LMICs [19] and informed by our discussions with stakeholders in Bangladesh [13], we formulated a topic guide to address the study's aims and objectives (**Table 1**). Our over-arching aim was to explore beliefs about CRDs and their management; the role and acceptability of PR; and any suggestions for implementation in Bangladesh. The topic guides were piloted to ensure that questions were asked using neutral language and that both positive and negative perceptions were probed.

**Table 1:** Topic guide

Objective	Questions and prompts
Understand participants’ beliefs about their CRD	Perceptions of their disease and how they were affected by it
	Beliefs about the cause and nature of their condition
Explore perceptions of pulmonary rehabilitation as part of their management	Acceptability of pulmonary rehabilitation as a treatment strategy (centre- and home-based)
	What benefits (if any) did they notice from the pulmonary rehabilitation?
	What challenges and barriers did they face?
	How did they overcome them?
	How did they feel about pulmonary rehabilitation before and after participating in the programme?
Gain insights into how pulmonary rehabilitation could be implemented	What changes would they recommend for future rehabilitation services?
	Would they recommend pulmonary rehabilitation to other CRD patients in their community?
Additional views	Any other additional thoughts they want to provide?

CRD – chronic respiratory disease

### Data collection and management

The interviews were conducted by NU, a male doctor with an interest in respiratory conditions and experience in conducting qualitative interviews, and MA, a male medical assistant who had completed four years of medical training. They were not involved in providing care at the clinic to avoid patients feeling hesitant to criticise their care providers. NU and MA had regular discussions about practical conduct of the interviews to maintain quality and consistency in data collection.

Due to the COVID-19 lockdown, the participants were typically interviewed via telephone at their preferred time. Each interview lasted approximately 45 minutes and was audio-recorded and transcribed verbatim in the original language by a Bengali-speaking transcriber employed by the Khulna pulmonary rehabilitation service. Transcripts were allocated a study ID, anonymised by the transcriber, and checked by the qualitative researchers who are both native Bengali speakers. We coded the transcripts in Bengali to preserve the nuances of the language and the full meaning of the interviews. Later, transcripts and codes were translated into English for sharing with English-speaking authors and for reproduction in publications. The audio recordings were destroyed at the end of the study.

### Data analysis

We used the grounded theory approach [20] in generating data as it originates inductively from the interview and addresses the research objectives as well as allowing additional themes (whether positive or negative) to emerge. Following the principles of grounded theory [21], two researchers (MH and NU) read and re-read the transcripts to familiarise themselves with the content before assigning codes to each sentence, phrase, or group of words. We then identified patterns among these codes to form initial themes, gradually developing both overarching themes and sub-themes. We then examined the relationship between the themes and sub-themes, reviewing, revising, and organising the provisional themes. This iterative process allowed us to generate the findings and draw inferences.

### Reflexivity

Throughout the study, we remained aware of the subjectivity that may arise due to personal experiences and current professional positions [22,23] (MH is an experienced clinician specialising in CRDs with a well-known intention to implement pulmonary rehabilitation in Bangladesh [13]). Although the interviews were conducted by researchers not involved with delivering care in the pulmonary rehabilitation centre, MH led the analysis and consciously strove to minimise the influence of his biases during the analysis process. The interview transcripts were anonymised to ensure that the coders’ knowledge of a participant’s clinical history did not influence their interpretation of the participant's statements during coding. The wider team were also involved in reviewing the coding and deliberating on the interpretation.

### Trustworthiness

We adopted approaches to enhance the reliability of the findings [24]. Members of the wider research team checked for conformability and accuracy of data analysis by examining excerpts from the data along with their corresponding codes, subcategories, and categories [25]. Additionally, we documented all the study steps to facilitate replication and verification by others. To establish transferability, we recruited a diverse range of participants through purposive sampling. MH and NU conducted duplicate coding and analysis separately, which increased dependability [26]. Disagreement was resolved by discussion; we engaged senior researchers from the University of Edinburgh if consensus was not achieved.

## RESULTS

We interviewed 15 patient participants; most completed the home-based pulmonary rehabilitation programmes, while two participants were selected from the pre-COVID-19 cohort enrolled in centre-based programme (**Table 2**). We ceased conducting interviews after we reached data saturation for the themes related to the three objectives.

**Table 2:** Participants’ characteristics

ID	Age in years	Sex	Distance in kilometres	Travel time in minutes	Diagnosis
Centre-1	61–70	F	10	20	COPD
Centre-2	41–50	F	25	45	ACOS
Home-3	21–30	M	31	45	PIAT
Home-7	41–50	F	160	240	Asthma
Home-10	61–70	M	6	15	COPD
Home-13	51–60	M	39	45	COPD
Home-14	61–70	M	66	75	Asthma
Home-17	41–50	M	15	20	Asthma
Home-21	61–70	M	66	120	COPD
Home-22	41–50	F	65	150	COPD
Home-23	51–60	M	87	300	PIAT
Home-28	31–40	F	15	30	Asthma
Home-39	61–70	M	7	30	COPD
Home-49	41–50	M	66	150	COPD
Home-50	41–50	M	60	110	COPD

ACOS – asthma COPD overlap syndrome, COPD – chronic obstructive pulmonary disease, F – female, M – male, PIAT – pulmonary impairment after tuberculosis

### Overview of the themes and sub-themes

We coded the data into three primary themes aligned with the objectives, subdividing each into sub-themes to explore the underlying patterns (**Table 3**). No additional themes emerged.

**Table 3:** Themes and sub-themes of qualitative analysis

Themes	Sub-themes
Understanding of CRDs	Naming their disease or describing the underlying disorder
	Causes of their disease
	Understanding the future of their disease
Perceptions of pulmonary rehabilitation	Understanding of pulmonary rehabilitation
	Benefits and challenges of pulmonary rehabilitation
	Overcoming the challenges
Implementation of pulmonary rehabilitation	Understanding the context
	Motivational strategy
	Developing resources and enhancing access to pulmonary rehabilitation

CRDs – chronic respiratory diseases

### Theme 1: Understanding of CRDs

#### Naming the disease or describing the underlying disorder

Clinical appointments in Bangladesh are short and most clinicians do not explain the diagnosis to the patient. Most patients described their illness by their current symptoms without exploring the disease process. They were bothered by their symptoms, and less concerned by the diagnostic label of their disease. In describing their understanding, they explained:

*I* have been suffering from cough, respiratory distress, and breathlessness on exertion for a long time. It was intolerable to me. *– Home-49: Male, 41–50 years, businessman with COPD*

This contrasted the typically asymptomatic, but well-known conditions such as hypertension and diabetes, which they defined as ‘diseases:’

(…) It’s only the cough, chest tightness, sleep disturbance and trouble with any physical effort for me. Otherwise, I don’t have any diseases like blood pressure, diabetes, etc. *– Home-21: Male, 61–70 years, a retired schoolteacher with COPD*

A few patients cited ‘asthma’ as their disease, even when that was not the cause of their symptoms; others described themselves as suffering from ‘Hapani’ – a Bengali term meaning breathlessness – which represents both asthma and COPD, even to the clinicians:

My problem was nothing more than breathlessness and difficulty in movement. Otherwise, I was quite well (…) My ‘Hapani’ developed after the treatment of TB [tuberculosis]. *– Home-3: Male, 21–30 years, businessman with PIAT*

#### Causes of their disease

Most participants discussed their understanding of how their disease started and developed. Some identified factors responsible for disease development, such as their occupation, history of childhood pneumonia, pulmonary tuberculosis, or family history of asthma:

I worked in a jute mill for 35 years with the exposure to huge dust that developed this problem. I leave work to get relief and feel better, but still, I can’t do hard physical work, or walk fast as before. *– Home-39: Male, 61–70 years, a retired jute mill worker with COPD*

#### Gaining insight into the future progression of their illness

Most participants discussed the natural history or course of the disease from their own experience. Others discussed the prognosis of the disease and the effect of treatment. Some patients derived confidence from witnessing other patients who had experienced positive outcomes from pulmonary rehabilitation, leading them to believe that they too would achieve similar results:

One of my neighbours had been suffering badly from cough, wheezing and breathlessness; he could not attend his workplace every day, now he is fine and doing all the things perfectly; he is cured. I am expecting such an outcome. *– Home-10: Male, 61–70 years, a wood seller with COPD*

In contrast, some were pessimistic and stated that they had no way of getting rid of their suffering but to live on drugs, as informed by another doctor:

I consulted one doctor who told me, your ‘Hapani’ (asthma) will never go away, you have to live with ‘Hapani’ and these drugs lifelong. *– Home-23: Male, 51–60 years, fisherman with PIAT*

### Theme 2: Perception of pulmonary rehabilitation

#### Knowledge about pulmonary rehabilitation

All participants discussed the pulmonary rehabilitation that they had experienced. Participation in a clinical study conducted by clinicians is poorly accepted in their context, and some expressed doubt about an ‘experimental intervention’ that may or may not help their disease because it was part of a research project:

I have never heard that exercise can alleviate breathlessness. This doctor is conducting research; let's see if it proves effective. *– Centre-2: Female, 40–50 years, housewife with asthma*

Meanwhile, some participants believed that, although pulmonary rehabilitation or increased levels of physical activity was ideal therapy for individuals with diabetes, it was not clear how it could help their respiratory symptoms:

I know people with diabetes walk every day as per the recommendation of their doctor. My husband told me that I have no diabetes, why does the doctor suggest you do exercise? But as my wise doctor suggested, I am continuing and getting benefits. *– Home-28: Female, 31–40 years, housewife, asthma*

#### Benefits and challenges of pulmonary rehabilitation

Most participants agreed pulmonary rehabilitation worked well after a few exercise sessions; some described how they had been able to resume their physical ability and mental well-being:

(…) When I came to sir (their doctor), it was horrible for me to think about climbing stairs due to ‘Hapani’. Now I can climb stairs up to the second floor, which I never thought (…)* – Home-50: Male, 41–50 years, businessman with COPD*

In-person supervision in centre-based rehabilitation gave people the confidence to engage in the exercise programme, and many were able to apply the strategies they had learnt from the centre therapists. Exercise-induced breathlessness, however, could be frightening when no one was there to help and caused one participant to stop home-based pulmonary rehabilitation:

According to sir’s (the doctor’s) suggestion, I started to exercise at home with the supervision of the girls (PR therapists); but during exercise, I got severe breathlessness which was very difficult for me to cope with. I took inhalers which did not work; I was so frightened that I stopped exercise (…) *– Home-10: Male 61–70 years, wood-seller with COPD*

Many participants discussed challenges in terms of time, facilities, money and family support. Some were living far away from the pulmonary rehabilitation centre with difficulty travelling or had commitments that precluded travel. ‘I have no way to leave home’ during harvest months’ (Home-7: Female, 41–50 years, farmer’s wife with asthma). A centre-based programme would be completely impossible for them:

It’s 75 kilometres from the pulmonary rehabilitation centre from my home, needs three to four hours to travel due to broken journey and poor transport service. How could I attend the centre twice a week for pulmonary rehabilitation at your centre? *– Home-13: Male, 51–60 years, meat seller with COPD*

In contrast, a few patients preferred centre-based pulmonary rehabilitation, citing several benefits, such as the opportunity to share views with people facing similar problems and a reduction in anxiety and depression:

When I join the exercise program with the group, I no longer feel alone. Moreover, during the workouts, there is an unspoken competition that arises when we see others exercising, which motivates us to push ourselves further and perform better. *– Centre-1: Female, 60–70 years, a retired schoolteacher with COPD*

#### Overcoming the challenges

Participants provided accounts of diverse strategies they employed to overcome their unique challenges. One participant even offered to arrange free local facilities in his area:

(…) I think home-based service is best for us, we can come to your centre once in two months and after learning from the centre we can practise it at home. You need to give us some pictures and video clips which we can follow. *– Home-49: Male, 41-50 years, businessman with COPD*

### Theme 3: Implementation of pulmonary rehabilitation

#### Understanding context

Nearly all participants agreed that, because pulmonary rehabilitation was introduced to Bangladesh relatively recently, the general population was largely unfamiliar with it; patients experienced a combination of hope and apprehension as they gradually understood that exercise was its fundamental aspect, despite breathlessness being a typical symptom of CRDs:

(…) I knew that physical movement made symptoms worse and I used to avoid movement as much I could. But my doctor told me exercise will help me to get rid of my problem. I have every trust in my doctor, as he is very knowledgeable and wise, I followed his suggestion, but I was always afraid of any exacerbation that could make me very sick. *– Home-7: Female, 41–50 years, schoolteacher with asthma*

Some family members tried to dissuade patients from believing it to be helpful:

(…) My husband said, ‘You can’t tolerate mild exertion, why are you to do exercise? Do you think it is safe for you?’ I think it may worsen your symptoms and you will be sick on exercise. *Home-28: Female, 31–40 years, housewife with asthma*

#### Motivational and awareness strategy

All participants emphasised the significance of increasing individual motivation and raising community awareness as key strategies for overcoming the prevailing health beliefs and other barriers in implementing pulmonary rehabilitation in Bangladesh:

(…) if people don’t know what [pulmonary rehabilitation] is, how it can help people, what is its financial benefit, how it will be implemented? (…) I think massive awareness among patients, people as well as healthcare providers is essential for the implementation of pulmonary rehabilitation. *– Home-23: Male, 51–60 years, Fisherman with PIAT*

#### Developing resources and enhancing access to pulmonary rehabilitation

To ensure the successful implementation and integration of pulmonary rehabilitation into routine clinical practice, participants highlighted two significant issues: The need for the training of qualified personnel and the importance of establishing easily accessible services:

(…) While I was doing exercise, one of our local doctors observed it and looked at the exercise booklet. He took it very positively and asked me where I got it from (…) he wanted to learn the techniques so that he can provide them to his patients. *– Home-14: Male, 61–70 years, a retired man with asthma*

Many participants described financial barriers to accessing the service, as some could not afford it despite the eligibility and their personal willingness:

You see, I am an old man, don’t have income and enough financial support from elsewhere. I trust your suggestion that exercise is good, but would anybody provide the service without money? I am struggling to buy drugs; how can I manage extra money for pulmonary rehabilitation? *– Home-39: Male, 61–70 years, ex-jute mill worker, COPD*

## DISCUSSION

The patients in our study perceived their disease through their experience of suffering, and the absence of such suffering was equated with a cure for the disease. Because breathlessness is exacerbated during exercise, many patients do not consider pulmonary rehabilitation an appropriate therapy for their condition. Therefore, they suggested that if it is recommended as part of their treatment, it should be thoroughly explained, emphasising its long-term advantages (including to their family) and creating widespread awareness among the public.

Participants highlighted many practical challenges with attending centre-based pulmonary rehabilitation. Home-based programmes overcame the need to travel, though exercise was not always possible at home because of domestic commitments. The successful implementation of pulmonary rehabilitation necessitates a multifaceted approach that considers the challenges faced by each patient and tailors strategies accordingly [14].

### Strengths and limitations

This study is the first in Bangladesh to explore the perceptions, beliefs, and attitudes of patients with CRDs regarding their disease and current evidence-based practices, including pulmonary rehabilitation. One of its strengths was the multidisciplinary team which included individuals with practical experience in delivering care in Bangladesh, as well as global experts in pulmonary rehabilitation and in implementation research, which provided diverse perspectives on the findings. More widely, this work was part of the pulmonary rehabilitation portfolio of the National Institutes of Health and Care Research Global Health Research Unit on Respiratory Health (RESPIRE) (PuRe: https://www.ed.ac.uk/usher/respire/research/non-communicable-diseases), which offered further opportunities for a balanced interpretation of the findings.

We were aware of and took steps to address several sources of bias. First, it was important that the interviewers were external to the pulmonary rehabilitation centre, so that participants felt free to share their opinions and express any negative experiences. This was achieved, although we cannot be sure that it completely overcame reticence to speak freely (both interviewers had clinical backgrounds and might, for example, have been regarded as part of the medical establishment). Likewise, strong relationships between pulmonary rehabilitation providers and patients might have discouraged some patients from offering negative comments. Second, the questions asked and themes pursued in the interviews were likely affected by the interviewers’ knowledge, background, and perceptions – a bias which we tried to mitigate by describing and remaining aware of their clinical background. Finally, the principal investigator who led the coding was the owner of the pulmonary rehabilitation centre and an advocate for the approach. We have declared this conflict of interest, and ensured that coding was reviewed by the wider research team to provide a broad multidisciplinary interpretation.

We conducted this study during the COVID-19 pandemic, when widespread restrictions across the country meant that conducting face-to-face interviews was not feasible. Videocalls were not usually feasible because of the limited or unstable technology infrastructure, especially in remote areas, and telephone calls meant we were unable to capture non-verbal information that would have been conveyed through in-person interactions. This likewise limited the field notes we could capture. Moreover, sometimes poor connectivity posed a challenge in clearly capturing all the voices, potentially resulting in the omission of important information.

The 15 interviews reached saturation for the research questions, enabling us to meet the key objectives. The enforced suspension of centre-based pulmonary rehabilitation meant that our findings were mainly from people who had completed home-based pulmonary rehabilitation, though we recruited two participants who had undertaken most of the face-to-face centre-based course and were able to comment from their perspective.

### Discussion in comparison with published findings

In marked disparity with the clinician’s biomedical understanding of the disease, individuals with CRDs develop their own unique explanation or ‘sense’ of their condition based on their personal experiences of suffering and sharing experiences with friends and family [27]. Patients are confronted with the challenge of understanding and managing a complex disease process in their context. Without an understanding of their condition, they are unable to take necessary measures to maintain their health or make informed decisions about their well-being [28,29], which affects their adherence to recommended management strategies [29]

The adoption of pulmonary rehabilitation poses a challenge for patients in Bangladesh, as it is a new intervention that leaves many of their questions unanswered. A systematic review of 48 studies conducted in a range of settings globally, concluded that factors such as context, environment, people's knowledge, and beliefs influenced uptake, attendance, and completion of pulmonary rehabilitation. [30]. We also found that the understanding of the disease process by families and friends can have a significant impact on the patient's acceptance of pulmonary rehabilitation. There is also evidence that acceptance of pulmonary rehabilitation is affected by capability-related factors, such as patients' cognitive abilities, understanding of the disease, and their ability to access the programme [29,31]. We did not formally assess cognition but identified multiple practical barriers that reduced the ability to adhere to a pulmonary rehabilitation regime.

Our participants had several suggestions for overcoming barriers, many of which resonate with strategies described in the literature as being conducive to reducing negative perceptions of pulmonary rehabilitation. These include, for example, educating patients and raising community awareness, adopting a personalised approach to cater for individual needs, empowering patients to actively participate in their own care, ensuring effective communication and support, promoting peer group support and networking, evaluating outcomes and collecting feedback, and fostering collaboration [32]. Implementation requires multiple strategies, including enhancing access to existing programmes, expanding the presence of such programmes within communities, establishing sustainable delivery models, securing reimbursement from payers, evaluating the cost-effectiveness, emphasising the sustainability of long-term outcomes, and identifying individuals for whom pulmonary rehabilitation is a priority [33,34].

Aside from prevailing health beliefs and patients' understanding of CRDs, the implementation of pulmonary rehabilitation in Bangladesh faces practical challenges, such as the accessibility and affordability of related services. Similar difficulties have been documented in numerous studies conducted in other regions [13,35-37]. Accessibility to pulmonary rehabilitation in Bangladesh is severely limited. Currently, we are aware of only three operational centres in the entire country, despite its population of approximately 160 million people. This scarcity poses significant challenges for individuals in accessing the services they need; additionally, the associated costs are excessively high. Bangladesh lacks dedicated institutional training programmes for pulmonary rehabilitation, resulting in a shortage of skilled therapists to support the care programmes. Additionally, there is a lack of awareness among potential referral sources, which leads to failure in identifying and referring eligible patients for pulmonary rehabilitation. Similar findings have been observed in other LMICs [38-40].

### Implications

We discovered that individuals often have their own perceptions of their medical condition, which may not always align with biomedical facts, leading to misconceptions about management. Breathlessness is frequently triggered by exertion, leading some patients to believe that the exercise component of pulmonary rehabilitation is making their condition worse. This underscores the importance of incorporating organised patient education, including information about the disease process, both in the pulmonary rehabilitation programme and in clinicians’ conversations with patients when recommending referrals. Providing examples of patients who have experienced improvement after participating in pulmonary rehabilitation may help address this concern. The influence of family, friends, and society on pulmonary rehabilitation implementation is significant, emphasising the need for ongoing stakeholder engagement.

Panic often arises from breathlessness attacks, causing patients to limit their daily activities. To address this issue, we recommend incorporating motivational interviews as part of the intervention. The local challenges were not unexpected, but were valuable reminders of specific barriers that need to be resolved before pulmonary rehabilitation can be rolled out in Bangladesh.

Our findings have implications for implementing pulmonary rehabilitation in Bangladesh; they outline a need to improve community awareness of respiratory diseases so that they are understood as ‘diseases’ in the same way as hypertension and diabetes (as opposed to the current perception of ‘Hapani’/symptoms). Patients and their families need to be supported to understand how an activity that causes short-term breathlessness can make them fitter and less breathless in the long term.

Pulmonary rehabilitation is a novel intervention in Bangladesh, and it is important to promote its widespread implementation throughout the country that can be tailored for delivery in various settings such as dedicated centres, community-based facilities, satellite programmes, or home-based approaches [14], depending on the specific needs and circumstances of the patients and their respective localities. Such innovations need robust evaluation, including a thorough cost-benefit analysis.

## CONCLUSIONS

This study sheds light on the complex challenges involved in managing patients with CRDs in Bangladesh. It emphasises that the patients' understanding of their condition significantly influences their acceptance and adoption of globally recommended management plans, regardless of the strength of the supporting evidence. Understanding how patients and their communities perceive their condition and the logistical barriers to acceptance is the first step to embedding this highly effective intervention into routine health care services in Bangladesh (and potentially more widely), with benefits for the increasing number of people living with CRDs both in Bangladesh and other LMICs.

## Additional material


Online Supplementary Document
