# Supplementary material for: Exploring the perceptions of patients with chronic respiratory diseases and their insights into pulmonary rehabilitation in Bangladesh
Source: J Glob Health. 2024 Feb 2;14:04036. doi: 10.7189/jogh.14.04036 (PMC10832548; doi:10.7189/jogh.14.04036)
Supplement: Online Supplementary Document [file jogh-14-04036-s001.pdf]

Online Supplementary Documents

Table S1. Standard for Reporting Qualitative Research (SRQR)

No	Topic	Item
Title and abstract		
S1	Title	Page 1
S2	Abstract	Page 2
Introduction		
S3	Problem formulation	Page 3
S4	Purpose or research question	Page 3-4
Methods		
S5	Qualitative approach and research paradigm	Page 3
S6	Research characteristics and reflexivity	Page 6
S7	Context	Page 3
S8	Sampling strategy	Page 4
S9	Ethical issue pertaining to human subjects	Page 4
S10	Data collection methods	Page 5
S11	Data collection instruments and technologies	Page 5
S12	Units of study	Page 4
S13	Data processing	Page 5-6
S14	Data analysis	Page 6
S15	Techniques to enhance trustworthiness	Page 6-7
Results and findings		
S16	Synthesis and interpretation	Page 7-12
S17	Links to empirical data	Page 7-12
Discussion		
S18	Integration with prior work, implications, transferability, and contribution (s) to the field	Page 14-15
S19	Limitations	Page 13
Other		
S20	Conflicts of interest	Page 17
S21	Funding	Page 17
